# The experiences of physiotherapists treating people with dementia who fracture their hip

**DOI:** 10.1186/s12877-017-0474-8

**Published:** 2017-04-20

**Authors:** AJ Hall, R Watkins, IA Lang, R Endacott, VA Goodwin

**Affiliations:** 1 NIHR CLAHRC South West Peninsula, University of Exeter Medical School, Exeter, UK; 2 School of Nursing and Midwifery (Faculty of Health and Human Sciences), Plymouth University, Plymouth, UK

**Keywords:** Physiotherapist, Physiotherapy, Dementia, Hip fracture, Experiences

## Abstract

**Background:**

It is estimated that people with dementia are approximately three times more likely to fracture their hip than sex and age matched controls. A report by the Chartered Society of Physiotherapy found that this population have poor access to rehabilitation as inpatients and in the community. A recent scoping review found a paucity of research in this area, indeed there has been no qualitative research undertaken with physiotherapists.

In order to address this evidence gap, the aim of this current study was to explore the experiences of physiotherapists treating this population.

**Methods:**

Semi-structured interviews with physiotherapists were undertaken in order to gain an in-depth understanding of how they manage this population. Physiotherapists were recruited from all over the UK and a purposive sampling strategy was employed. Thematic analysis was utilised.

**Results:**

A total of 12 physiotherapists were interviewed, at which stage data saturation was reached as no new themes were emerging. The participants had a broad range of experience both in physical and mental health settings. Analysis identified three separate themes: challenges, “thinking outside the box” and realising potential.

Physiotherapists felt significant pressures and challenges regarding many aspects of the management of this population. Mainly this was the result of pressures placed on them by guidelines and targets that may not be achievable or appropriate for those with dementia. The challenges and importance of risk taking was also highlighted for this population with an appreciation that standard treatment techniques may need adapting. “Rehabilitation potential” was highlighted as an important consideration, but challenging to determine.

**Conclusion:**

Interventions for the management of people with dementia and hip fracture need to consider that a traditional biomedical physiotherapy approach may not be the most appropriate approach to use with this population. However physiotherapists reported feeling pressurised to conform to a biomedical approach.

**Electronic supplementary material:**

The online version of this article (doi:10.1186/s12877-017-0474-8) contains supplementary material, which is available to authorized users.

## Background

The recovery of people following a hip fracture is often complex and involves a variety of factors such as physical, psychological and social components [1], indeed, it is suggested that only 40–60% of people who sustain a hip fracture recover their pre-fracture level of mobility and ability to perform activities of daily living [2]. Hip fractures are the third most common cause of admission into an acute setting [3] and lead to high levels of mortality [4] and morbidity [5]. The management of hip fractures can be more challenging if patients have dementia. It is estimated that 40% of people who fracture their hip will have coexisting dementia [6], which equates to approximately 32,000 of the 80,000 [6] people who sustain a hip fracture per year in the UK.

The majority of people who fracture their hip will undergo a surgical intervention to repair the fracture, the type of which will vary according to multiple factors including type of fracture and other co-morbidities. NICE guidelines recommend that the person should be seen by a physiotherapist within 24 h of surgery [7] and with an estimated 80,000 people fracturing their hip each year [6] this represents a significant contribution of people with hip fracture to a physiotherapists’ caseload.

A previous scoping review [8] has highlighted the lack of evidence to guide physiotherapists on how to manage people with dementia who fracture their hip, as well as a lack of qualitative research to explore the experiences of physiotherapists delivering a physiotherapy intervention to this population. It is suggested that this population are often poorly managed [9] which is unsurprising in view of the paucity of evidence to guide interventions. The lack of qualitative research in this area is in line with physiotherapy in general [10] and it is suggested that until recently few physiotherapy researchers had shown interest in qualitative work [9].

In light of the lack of qualitative research in this area, the aim of this current study was to explore the experiences of a range of physiotherapists treating this population, any difficulties they face and how they may overcome these.

## Methods

Semi-structured, face to face interviews with a range of physiotherapists working for the National Health Service (NHS) in the UK were undertaken in order to gain in-depth understanding of physiotherapists’ experiences of treating people with dementia who fracture their hip. A qualitative approach was used as it enabled in depth exploration of participants experiences and perspectives.

### Participants

Physiotherapists were recruited from throughout the UK and a purposive sampling strategy was employed based upon inclusion of participants with a variety of different experiences, knowledge and demographics. It was deemed important to get a variety of clinicians with different levels of expertise in treating this population as this would reflect clinical settings. An advert was placed on the Interactive Chartered Society of Physiotherapy (iCSP) website and personal contacts of the authors were contacted for potential involvement. The sampling sought to recruit participants with a range of characteristics including a range of experience, gender, speciality and setting in which they worked. Recruitment and interviewing continued until data saturation was reached and no new themes were emerging.

### Data collection and analysis

An interview topic guide (see Additional file 1) was used to guide the interview process. The questions were flexible, open-ended and broad, while focused on the topic in order to elicit rich responses from participants [11]. Participants were asked the same initial questions, but the questions were worded so that responses were open-ended allowing the participant to describe their experience in their own words [12]. The interviews explored the experiences of physiotherapists treating people with dementia who fracture their hip, the techniques they used and any difficulties they may face. The interviewer (AH) was a physiotherapist which allowed a deep discussion regarding physiotherapy interventions. In order to ensure any potential bias from the interviewer sharing the same professional background as the participants, a second researcher who was not a physiotherapist would independently code the data. The interviews were face to face and lasted approximately 45 min, they were audio recorded and transcribed verbatim. Development of themes and the process of purposeful sampling was aided by the usage of memos [11] throughout the data collection and analysis. Memos taken during data collection enabled the author to determine characteristics of participants that were important to gain further insight into potential themes. Furthermore, these memos were used during discussion with the other authors [13] about emerging themes.

Two of the authors (AH and RW) independently coded the transcripts. The two authors discussed and compared coding strategies and resolved any disagreements. NVivo 11 (QSR International) was used to organize and code the data and allowed a process of thematic analysis to be undertaken. The process of thematic analysis was guided by the methods suggested by Braun and Clarke [14]. The initial stage of searching for themes began during data collection and continued throughout the whole data collection stage. Initial coding followed data collection and codes were organised into preliminary themes. These preliminary themes were structured into a thematic map and were further refined while reviewing these themes throughout the data analysis.

In order to increase the internal validity of the results, a process of analyst triangulation [15] was undertaken. This involved the researchers independently analysing a selection of the coded data and then findings were compared. The process of thematic development was aided by a process of peer debriefing [16] whereby deduced themes were discussed amongst the authors to ensure trustworthiness of the analysis.

## Results

A total of 12 physiotherapists working in the National Health Service in the UK were interviewed, at which point no new data was emerging so data saturation had seemingly been reached. The participants represented a broad range of experiences both in physical and mental health settings (see Table 1).

**Table 1:** - Participant characteristics

Number of participants (*n* = 12)		
Location	South East	2
	Midlands	2
	North West	1
	North East	4
	Wales	1
	London	2
UK Pay Scale	6	2
	7	8
	8+	2
Gender	Male	3
	Female	9
Years of Experience	0–5	1
	5–9	1
	10–19	6
	20–29	3
	30+	1
Specialty	Physical Health	6
	Mental Health	5
	Both	1
Primary Location	Community	5
	In-patient	4
	Out-patient	1
	Mixed	2

Thematic analysis identified three separate themes emerging from the data. There were significant challenges reported by physiotherapists, leading to the need to “think outside the box” to develop management strategies for this population. The final theme draws on positive experiences of treating this population and the importance of appreciation of the concept of “rehabilitation potential”.

### Challenges

One of the most significant themes involved the challenges felt by physiotherapists regarding various aspects of the patients’ management. These challenges focused on the poor attitudes of others, lack of services for this population and also the importance of medical management, all of which had a significant impact on the ability to undertake physiotherapy. These challenges were reported universally in all settings and amongst different specialities, but the challenges face varied in their nature.

#### Frustrations

Several of the physiotherapists suggested that less experienced physiotherapists demonstrated a significant fear and panic of treating people with dementia *“they have a fear of dementia so I think they think “panic” and they don’t understand” (acute, mental health).* It was also reported by several of the participants that the presence of dementia was as often being an excuse not to treat the patient or as a cause of poor outcomes.

*“its always put as a limiting factor “ooh, they’re doing ok, but they have got dementia so they won’t go much further” or …. “they’re not doing very well, it’s ‘cos they’ve got dementia.” (community, physical health)*

People with dementia were described as regularly being *“written off far too early” (community, physical health)* after suffering a hip fracture, frequently without valid reason, but potentially the result of lack of knowledge or experience of treating people with dementia rather than for true physiological reasons. Community based physiotherapists felt that patients were often judged for their potential to improve in an acute setting which was an inappropriate setting to provide physiotherapy. This often prevented them being referred to community based services. Acute physiotherapists also acknowledged the acute setting as being inappropriate for this population, but felt there was no option as they were frequently unable to refer to community based services.

A further frustration reported was a lack of availability of services for people with dementia. It was reported that a diagnosis of dementia could exclude people from accessing some services.

*“so do they ‘qualify’ for an intermediate care bed if they [are deemed to] have no rehab potential?” (Community, physical health)*

Long community waiting lists and complex referral pathways were reported by acute physiotherapists to delay discharge from acute settings. There was a universal concern that patients needed to be seen quickly post hip fracture, as delays in offering treatment resulted in poorer outcomes.

However, a concerning suggestion made by community based physiotherapists was that often people with dementia and hip fracture are not referred for ongoing physiotherapy following discharge from acute settings. This was supported by acute physiotherapists highlighting difficulty referring to acute services, or referrals getting lost in complicated referral processes. Physiotherapists in the community reported being aware of people not being referred for ongoing input with some reporting actively seeking out patients themselves.

*“[he] wasn’t referred to me, I came across [him] shall we say. I’d heard stories…..” [Community, physical health]*

The majority of physiotherapists reported a pressure to prove effectiveness of their interventions and to justify the amount of input they provided, in all settings and specialities. Physiotherapists reported that standardised outcome measures were not appropriate for this population; however there was a general feeling amongst all that there was a need to use some form of outcome measure.

*“How can you actually say that a treatment is effective in the absence of an internationally validated outcome measure such as Tinetti or Berg…..” (Community, physical health)*

Acute physiotherapists suggested that national guidelines pose unachievable targets for physiotherapists to achieve when treating this population in view of resource limitations and commonly occuring post-operative complications such as delirium.

*“You know the NICE guidelines are suggesting that it’s very important to get these people moving as early as possible so there is a recommendation …… early mobilisation within 24 hours……. which is a ridiculous recommendation for this population anyway.” (In-patient, physical health)*

#### Medical management

The importance of good medical management of this population was mentioned by all physiotherapists in different contexts. Analgesia was reported to be vital in order to be able to offer effective physiotherapy, however this was often inadequate or inappropriate. It was commonly felt that analgesia should be routinely given, especially in the acute stages, to allow physiotherapy to be undertaken.

*“All our patients are in pain and most of the things we’re going to ask them to do acutely is going to be painful” (In-patient, physical health)*

Further medical issues such as nutritional needs of the patient were described as being vital to ensure physiotherapy can be undertaken, however it was felt that frequently the importance of this was overlooked. Post-operative delirium was reported to hinder the acute physiotherapy and was frequently poorly managed or diagnosed in people with dementia.

#### Role of collaboration

Few of the physiotherapists talked about involving doctors in the physiotherapy process in a positive manner. There was a perceived challenge of working with orthopaedic doctors whose biomedical approach to the treatment of this population did not necessarily fit with physiotherapy priorities. However, close working with occupational therapists (OTs) was reported by several of the physiotherapists, especially in in-patient settings.

*“But all my best outcomes in terms of mental health, trauma and orthopaedics, burns, any area, it has always been a good MDT approach with OT’s” (In-patient, mental health)*

Low physiotherapy staffing levels was reported frequently. The use of physiotherapy assistants, which were recognised to be a more cost effective approach to treating this population, was adopted variably. Some services preferred to employ qualified staff, whereas others found them invaluable; *“they are worth their weight in gold” (In-patient, mental health).* Where they were used, they were used to provide extra support during mobility re-training, continue exercise programmes that had been started by the physiotherapist, or undertake functional tasks such as outdoor mobility.

Involving family and carers in physiotherapy was discussed by all physiotherapists. The majority were keen to have family and carers involved in the treatments – asking them to continue exercises at home, or provide background information. However, some reported that carers often had too high levels of stress and carer burden to be able to be involved, or were too elderly or infirm themselves.

*“it’s an extra task that we’re asking them to do in a very stressful carers situation.” (Outpatient, physical health)*

#### Biomedical v’s person centred care approach

Physiotherapists suggested feeling pressured to comply with unsuitable biomedical assessments and outcomes; however this was the only approach that was taught at undergraduate level. Mental health physiotherapists reported that such biomedical approaches simply were ineffective for this population which led to them needing to change the way they manage this population. The adoption of person-centred care approaches were described commonly by physiotherapists working in a variety of specialities.

*“Do they like fishing? Do they like football? Do they like – then let’s go see a match. Maybe the rehab is walking to a football match? Maybe rehab is walking to the football ground?” (Outpatient, physical health)*

### Thinking outside the box

It was universally felt that interventions needed to be adapted for people who had dementia compared to those without, incorporating this person-centred care approach to treatment. The ability to adapt interventions was dependant on the availability of resources and time. Physiotherapists working in mental health settings generally described more novel techniques such as taking patients to community facilities such as boxing gyms to adapt interventions.

#### “Classic approaches just go out the window”

Despite being one of the most prevalent symptoms of dementia, interestingly very few physiotherapists suggested using any specific strategies to overcome memory difficulties, with only one recommended using a memory book, or written instructions, to enable them patients continue exercises independently. Instead, the majority of physiotherapists described pragmatic approaches to overcome memory problems. These techniques revolved around frequently prompting patients and adapting quantity and frequency of treatments.

*“they couldn’t sustain any effort for very long anyway but to get them three, four, five times a day rather than one twenty minute session made much more sense” (In-patient, physical health)*

The importance of a consistent physiotherapist treating the patient was highlighted alongside creating a regular daily routine to try and reduce disorientation. The majority of physiotherapists also reported the importance of the environment on the patients’ physiotherapy, with a variety of opinions; however, it was commonly felt that a familiar environment (typically home) was the most suitable.

*“I think some are patients being assessed in the wrong environment as if they are in a very unfamiliar situation and environment, then perhaps you don’t see the potential” (Out-patient, physical health)*

Physiotherapists in all settings reported that a lot of behavioural difficulties were avoided by ensuring appropriate communication. Taking time to build a relationship was reported to be vital as well as allowing the patient to lead movement. Physical contact with the patient was deemed appropriate to build a relationship, but during treatments this was not necessarily beneficial.

*“But when we stand them, we start to take away that support slowly so that people don’t become dependent and we do try and keep hands off, because I think with a lot of our patients, you put lots of hands on, it’s giving a lot of sensory input to them and they think “great and I’ll just lean back on it”” (In-patient, mental health)*

Adapting verbal communication was suggested such as breaking down instructions, using short sentences and speaking more slowly. The importance of not overloading a person with verbal input was also reported by several physiotherapists.

*“I think we’re all for wanting to give people as much information as they need and overwhelming them sometimes with information and some of the time it’s about taking a step back.” (In-patient, mental health)*

The importance of non-verbal communication was reported by many of the mental health physiotherapists including the importance of body positioning and allowing the person to see their face. This helped the person communicate, but also was felt to reduce some behavioural difficulties such as aggression.

Low mood or motivation was suggested to reduce engagement in physiotherapy sessions. Several physiotherapists reported including their patient in group rehabilitation to try and improve mood and therefore engagement. Physiotherapists working in community settings reported trying to determine the activities that a person previously enjoyed, trying to engage them in such activities and incorporating physiotherapy into this. However, this was very dependent on the time and resources available.

*“Certainly know their life history. Certainly know their story. What makes sense to them? What is their context? What is their environment? What are they used to? So that you can interpret what they’re saying….” (Outpatient, physical health)*

Encouraging the patient to set their own goals if able was deemed important in less acute settings. Physiotherapists tried to adapt interventions to make them meaningful to the patient and enjoyable. This required a significant time investment to learn about the patient and spend time talking to them and their relatives. In acute settings, the goals revolved around discharge planning.

#### Challenges of taking risks

Acute physiotherapists did not report ‘risk taking’ as being a problem, however community based physiotherapists reported it as having a significant impact on their clinical reasoning and management of the patient. Physiotherapy involves challenging a person’s physical ability and this, by its very nature, increases the risk of further physical injury. Reported risks included allowing a person to walk without a walking aid, or allowing them to return home with a high risk of falls. However, the extent to which a physiotherapist is prepared to accept this risk was suggested to affect the person’s potential to improve. It was felt that some physiotherapists are reluctant to take any risks.

*“I think people are frightened of litigation and getting into trouble for things” (In-patient, mental health)*

It was described in the acute setting that patients often have their mobility deliberately restricted to try and prevent the risk of further falls. This was in the form of using bed rails, sedatives or discouraging people trying to stand or walk. However, physiotherapists recognised that this was disabling people further, made physiotherapy progress slower and increased risks such as chest infections, pressure sores and cardiovascular complications. Therefore their aim was to promote mobility, while accepting that imperfect treatments were sometimes necessary. Community and mental health physiotherapists felt that avoiding risk limited the person’s ability to live a fulfilled life.

*“You’re not necessarily having less risk by doing something more protective if you think about what you’re doing for that patient and you’re you know you’re de-conditioning them and you’re disabling them aren’t you?” (In-patient, mental health)*

The ability to take positive risks by the physiotherapist was reported to be associated with confidence and experience of that clinician and was learnt implicitly. This was particularly evident around issues of capacity and consent, where physiotherapists felt treating people without explicit consent was a challenge.

#### Value of experience

The importance of clinician’s experience was discussed to varying extents. There were different methods of gaining experience, with the majority of physiotherapists relying on experiential or implicit learning, with only small amounts learnt explicitly. All participants reported a significant lack of undergraduate education around dementia, instead relying on post-graduate experience.

*“Experience! Trial and error! Did I learn about dementia during my undergraduate training? No!“ (Community, mental health)*

Few physical health physiotherapists had undertaken any training in dementia, despite having sought it. The physiotherapists working in mental health settings had accessed more training in dementia care, but some still felt there was a lack of explicit education available. Some of the participants sought evidence, but felt that little advancement had been made in the literature in recent years, therefore used research sparingly.

*“Yes, let’s look at the research. You know, yes, let’s be informed by it but let’s not be dictated to by it.” (outpatient, physical health)*

When questioned physiotherapist struggled to explain how they had learnt to treat this population. Methods of “trial and error” were frequently reported – more so amongst more experienced physiotherapists. There was an element of assumed translation from personal life experiences, other physiotherapy experiences and a general feeling of compassion towards their patients which assisted their management.

*“It’s really hard to describe really, how you know……. you just get a feel for it, which is hard to define.” (in-patient, mental health)*

### Realising potential

Determining potential was a challenge reported by all physiotherapists, with the term “rehabilitation potential” frequently being used in this context. This is a label that was reported to be used mainly in the acute setting, to classify whether somebody has the potential to improve physically. There was significant disagreement about the value of this label.

Generally in-patient physiotherapists found this label useful as it helped determine the patient’s pathway, although they recognised that it was often poorly used and needed to be justified. Physiotherapists working in mental health and community settings viewed this term less favourably, reporting that it was often applied to a patient too soon and could be very detrimental to future services that were offered to that patient.

*“I see a lot of negative labels being used for people with dementia and once that label has been put on, it’s almost like they can’t get rid of it” (in-patient, mental health)*

Mental health physiotherapists further argued that applying such a label to a patient was due to a failing of the physiotherapist or the service provision rather than actual physical potential the patient has.

*“I believe everyone has rehab potential if you are skilled enough, have enough time and resources to be able to objectively work on a person’s goal.” (in-patient, mental health)*

Furthermore, it was suggested that such potential was often prejudged; assuming people with dementia could not be rehabilitated and therefore not even attempting to engage them in physiotherapy.

*“They’d looked at her page and before I’d even questioned them about why they’d not got her out of bed they’d already said she’d got no rehab potential.” (in-patient, mental health)*

Despite lots of negative feelings expressed towards the management of this population, various physiotherapists reported positive experiences. It was suggested by several physiotherapists that the presence of dementia after hip fracture was not necessarily a problem.

***“*** *sometimes the dementia can actually be to an advantage because the person has difficulty remembering the fact that they have fractured their hip” (In-patient, mental health)*

There was general consensus that there was variability in outcomes, but there were many examples of positive outcomes following hip fracture. Such positive examples were used by the physiotherapists to educate other healthcare professionals that people with dementia could recover following hip fracture.

***“*** *We’ve had some really positive outcomes and that in itself breeds positivity.” (In-patient, mental health)*

Several physiotherapists expressed a real passion for treating people with dementia and hip fracture including the satisfaction of achieving success in what was universally felt to be a challenging population.

*“I really enjoy it and I enjoy the successes of people with dementia going home.” (Mental health, acute)*

Moreover, the differing techniques used have been suggested to increase holistic approaches to treatment and increase creativity. These skills were reportedly directly transferable to other areas of physiotherapy. This was seen as being important for junior staff that may be rotating through a variety of different specialities.

Physiotherapists reported frequently acting as advocates for their patients, in order to ensure effective management where they felt treatment was sub-optimum. They felt they were ideally placed to co-ordinate their management.

*“I think giving the dementia patients a voice and being advocates for them rather than them just being dismissed” (In-patient, mental health)*

## Discussion

The aims of this study were to explore the experiences of physiotherapists who treat people with dementia and hip fracture. We interviewed 12 physiotherapists working in the UK within a variety of different healthcare settings and roles, identifying three main themes and a further nine subthemes.

This study discusses experiences of those people with dementia who get referred for physiotherapy following hip fracture, reporting the challenges faced by the physiotherapists treating this population. Furthermore, it also highlighted the difficulty physiotherapist’s face trying to refer patients to other services – specifically community based services, suggesting a lack of care pathways for this population could negatively affect their rehabilitation journey. This reflects a survey undertaken by the Chartered Society of Physiotherapy in conjunction with British Orthopaedic Association [17], which suggested that less than half of people with dementia and hip fracture get referred for community based follow-up. A recent retrospective cohort study suggested similar figures, reporting 40.1% of people with dementia did not receive any physiotherapy following hip fracture [18]. This highlights a large population of people who are not in receipt of physiotherapy and whose outcomes remain unknown. It has been highlighted that there is a the need for greater research into the effectiveness of care pathways for patients with dementia [19] and clarification about the definition of the term. Lack of care pathways being used in the management of people with dementia could be due to difficulty developing such pathways due to uncertainty surrounding aspects of dementia such as diagnosis and disease trajectory. However, this appears to pose a challenge to physiotherapists when managing this population.

Comparable experiences of physiotherapists to those in our study were found in neurological and palliative care specialities. Findings from a study using semi-structured interviews [20] with 11 physiotherapists working in various palliative care settings demonstrated that the participants felt that their role was to maximise independence and improve quality of life. They identified similar barriers and enablers in the form of communication, resources, teamwork, and training. This could suggest that the challenges faced by physiotherapists treating people with hip fracture and dementia may be similar to those faced by physiotherapists working with palliative patients and perhaps reflects the challenges of managing a person with dementia who fractures their hip. However, the fundamental difference in treating these populations is the difficulty in engaging and communicating with the person with dementia. Our participants described a variety of approaches of trying to overcome these difficulties, but these were very dependent on available resources such as time and staffing. Collaboration and communication with other services and healthcare professionals was reported variably. Involvement of carers and relatives was reported unanimously as being imperative, however, involving a wider multi-disciplinary team was reported to often be challenging. A recent government report [21] highlighted this fragmented approach to the care of patients with dementia.

Pressures placed upon physiotherapists by national guidelines, commissioners and managers were a common source of frustration for our participants. The validity of guidelines such as early mobilisation was questioned by some of our participants, but the suggestion was that the guidelines were not achievable due to resource limitations and service pressures, rather than a patient’s physiological ability or deficient skills of the physiotherapist. This is supported by a recent qualitative study which ascertained that barriers for referral for rehabilitation were the availability of human and physical resources [22].

Resource pressures, in conjunction with others lack of knowledge, were suggested to limit the physical potential of a population in a study similar to ours, conducted in the neurological field [23]. “Rehabilitation potential” was a term mentioned by many of our participants and was deemed to be challenging to determine in people with dementia and hip fracture. A literature review [24] found only a small number of studies on this topic, reporting that the methodological quality of the papers was insufficient to undertake a formal systematic review, favouring a thematic synthesis, the results of which suggested that there was insufficient evidence as how to determine a person’s rehabilitation potential. The lack of evidence in the literature surrounding this label is in contrast to the reported importance of this term when attempting to determine patient pathways. However, the importance of this label was inconsistent amongst our participants.

In support of previous research [25], an apparent challenge physiotherapists described was the need to employ person-centred approaches to their treatment, determined by an appreciation that standard treatment techniques were not effective if used without consideration for the effects of the dementia. However, this was a challenge working within a health paradigm which is biomedical in its background. An auto ethnographic study [26] investigated two physiotherapists working in neurological rehabilitation supports this theory and further suggested the importance of moving away from a biomedical approach that is commonplace for physiotherapists. The importance and challenges of “risk taking” was evident, but more experienced physiotherapists felt able to manage this element of the patient’s treatment. The avoidance of such risk has been suggested to be against the aim of ‘inclusion’ which is deemed to be what people with dementia and their families strive to achieve [27], forming a fundamental component of citizenship.

Ensuring their practice was evidence based represented a challenge to some of our participants due to the lack of published research into the management of this population in combination with the resource pressures. Where there was little published research to inform practice, physiotherapists described using their clinical expertise and experience in order to treat this population. This is the fundamental basis of evidence based practice, whereby there is a required integration of scientific evidence, clinical experiences and patients values and experiences [28]. The value of experience was highlighted by the majority of our participants in view of the lack of evidence available.

This is the first qualitative study looking at in-depth experiences of physiotherapists treating people with dementia who fracture their hip. One previous study looked at the difficulties healthcare professionals faced treating this population, employing a questionnaire to determine what cognitive deficits health care professionals, including physiotherapists, found most difficult to manage [29]. Analogous to our study, they found that health care professionals perceive memory impairment, lack of insight and inability to carry out purposeful movement to be main barriers to rehabilitation for this population. Our participants described a variety of different techniques and methods aimed at trying to overcome these deficits, however, the lack of knowledge and service pressures were reported to significantly affect the ability to practice the techniques. This lead to wide variations in the physiotherapy offered to patients. Only one further qualitative study involved a single physiotherapist amongst other healthcare professionals explored the decision making around accepting patients with dementia into rehabilitation following hip fracture. They described a unanimous belief that decisions about access to rehabilitation should be based on the ability to participate in rehabilitation and not solely on cognitive ability.

### Limitations

The fact that the primary researcher is a physiotherapist could be considered a strength of the study, or conversely a weakness. Controversy about the effect of “insider versus outsider” interviewers is significant. However, as suggested by Hockey [30], the advantage could be considered the *“lack of culture shock or disorientation, the possibility of enhanced rapport and communication [and] the ability to gauge the honesty and accuracy of responses”* [30], p 199). In order to ensure that this did not introduce any potential bias to the study, the second reviewer who coded the transcripts was a health researcher, but not a physiotherapist, nor had any specific experience of working with physiotherapists.

The other weakness of this study is the limitation to interviewing only physiotherapists. We acknowledge that the treatment of this population rarely involves physiotherapy in isolation, frequently involving a variety of services and professionals. However, this study aimed to explore the experiences only of physiotherapists, but future work looking at the experiences of other professionals may add to the understanding of rehabilitation of this population further.

## Conclusion

For patients who are referred for physiotherapy, this study provides some interesting insights into the experiences and difficulties that physiotherapists face treating people with dementia after hip fracture. It was commonly felt that the presence of dementia meant that people could not be treated using the same treatments and methods as people without. Therefore it was suggested that the guidelines need to reflect this in order to reduce pressures on physiotherapists to accomplish targets which are unachievable in the presence of dementia. The results support concerns raised by the Chartered Society of Physiotherapy and the British Orthopaedic Association and suggests an urgent requirement for the physiotherapy management of this population to be reconsidered.

However, the aptitude of physiotherapists treating this population has led to potential development of physiotherapists working in new roles, such as mental health liaison and fragility specialist roles. The experience and expertise of physiotherapists has a significant value to add to such a role, despite it being routinely carried out by nurses.

This study addressed the experiences of physiotherapists delivering care to this population, but did not seek to explore the experiences of receiving the intervention. Therefore, further work is planned to explore the experiences of people receiving physiotherapy when the person has dementia –from the person with dementia and also the carers’ perspectives.

## Additional file

Additional file 1: Interview topic guide. This document is the topic guide used by the researcher to structure all participant interviews. (DOCX 15 kb)

## Abbreviations

Icsp: Interactive Chartered Society of Physiotherapy
MDT: multi-disciplinary team
NICE: National Institute of Clinical Excellence
OT: occupational therapist
