# Supplementary material for: The experiences of physiotherapists treating people with dementia who fracture their hip
Source: BMC Geriatr. 2017 Apr 20;17:91. doi: 10.1186/s12877-017-0474-8 (PMC5399424; doi:10.1186/s12877-017-0474-8)
Supplement: Additional file 1: — Interview topic guide. This document is the topic guide used by the researcher to structure all participant interviews. (DOCX 15 kb) [file 12877_2017_474_MOESM1_ESM.docx]

**INTERVIEW TOPIC GUIDE**

***“The experiences of physiotherapists treating people with dementia who fracture their hip”***

**[Version 1.1]**

**1, Gain Background**

“Can you describe your experiences of treating people with dementia who fracture their hip?”

Explore;

- Recent examples
- Attitudes to rehabilitation
- Positive/negative

**2, Techniques/strategies used**

“Can you explain how you treat people with hip dementia who fracture their hip?”

Explore;

- Functional techniques
- Psychological approaches
- Motivation
- Support strategies
- Use of verbal/visual prompts
- Variation from people without dementia
- Examples of techniques which have/haven’t worked
- Specific training undertaken
- Tacit knowledge

**3, Rehabilitation potential**

“What influences your decision about whether you offer these people rehabilitation?

Explore;

- Rehab v’s no rehab
- Length of programme
- Frequency
- Cognitive level of person
- Compliance
- Pre-morbid functional ability

**4, Others involved in the rehabilitation**

“What do you feel are the benefits and challenges of getting carers and support workers involved in the rehabilitation?”

Explore;

- Role of carers
- Do people work well with their carers
- Are carers keen to be involved
- **Role of support workers**
- Training of support workers

**5, Outcomes**

“How do you measure success in these people?”

Explore;

- Outcome measures
- Functional
- Physical
- Participation
- Quality of life
- Carer burden
